# The role of disease characteristics in the ethical debate on personal genome testing

**DOI:** 10.1186/1755-8794-5-4

**Published:** 2012-01-19

**Authors:** Eline M Bunnik, Maartje HN Schermer, A Cecile JW Janssens

**Affiliations:** 1 Dept. of Medical Ethics and Philosophy of Medicine, Erasmus University Medical Center, dr. Molewaterplein 50, Rotterdam, 3015 GE, the Netherlands; 2 Dept. of Epidemiology, Erasmus University Medical Center, dr. Molewaterplein 50, Rotterdam, 3015 GE, the Netherlands

## Abstract

**Background:**

Companies are currently marketing personal genome tests directly-to-consumer that provide genetic susceptibility testing for a range of multifactorial diseases simultaneously. As these tests comprise multiple risk analyses for multiple diseases, they may be difficult to evaluate. Insight into morally relevant differences between diseases will assist researchers, healthcare professionals, policy-makers and other stakeholders in the ethical evaluation of personal genome tests.

**Discussion:**

In this paper, we identify and discuss four disease characteristics - severity, actionability, age of onset, and the somatic/psychiatric nature of disease - and show how these lead to specific ethical issues. By way of illustration, we apply this framework to genetic susceptibility testing for three diseases: type 2 diabetes, age-related macular degeneration and clinical depression. For these three diseases, we point out the ethical issues that are relevant to the question whether it is morally justifiable to offer genetic susceptibility testing to adults or to children or minors, and on what conditions.

**Summary:**

We conclude that the ethical evaluation of personal genome tests is challenging, for the ethical issues differ with the diseases tested for. An understanding of the ethical significance of disease characteristics will improve the ethical, legal and societal debate on personal genome testing.

## Background

A growing number of personal genome testing services are presently available that estimate genetic susceptibility to multifactorial diseases [1]. Unlike genetic tests for monogenic diseases, susceptibility tests for multifactorial diseases can be obtained almost exclusively on the direct-to-consumer market. Personal genome tests are non-targeted: they determine a person's risk for a multitude of multifactorial diseases simultaneously [2-5], including cardiovascular disease, Alzheimer's disease, osteoporosis, type 2 diabetes, schizophrenia, and many types of cancer. One of the leading personal genome testing companies is currently estimating personal risks for over two hundred diseases and other phenotypic traits in one single test [2]. The large quantity of test results will have limited clinical significance for and varying emotional impact on consumers, which are in part connected with differences between the diseases tested for.

Differences between diseases may have important implications for the ethical, legal and societal debate on genetic susceptibility testing for multifactorial diseases (for the remainder of this paper we will use the term 'susceptibility testing'), whether it is offered as part of a personal genome test or on its own, for a single disease, whether within the clinic or on the direct-to-consumer market. The offering, for instance, of susceptibility testing for less severe diseases for which there are preventive options available may be morally acceptable, whereas the same test for severe diseases in the absence of treatment options may not. Personal genome tests may comprise both severe and less severe diseases and may as a whole thus be difficult to evaluate. In recent years, a range of ethical, legal and societal issues has been touched upon in professional and academic discussions on genetic testing, such as privacy issues, psychological impact, regulatory issues, and informed consent [6-8]. These issues however may not arise in all susceptibility tests that are offered within one personal genome test. Privacy issues, for example, may be more pressing for genetic tests for psychiatric diseases than for somatic diseases, and the psychological impact of testing may be more serious in severe diseases than in less severe diseases. With this paper, we propose a systematic approach to the ethical evaluation of susceptibility testing, which considers differences between diseases and which explains what ethical issues are to be addressed, on the basis of disease characteristics.

As in this paper we focus explicitly on *disease *characteristics and their impact on the ethical debate, we do not elaborate upon characteristics of the *test*, such as clinical validity and clinical utility. Genetic susceptibility tests differ from presymptomatic tests for monogenic diseases: they indicate risk rather than diagnosis and are generally of limited to moderate clinical validity and utility. Clinical validity and utility are of paramount importance to the ethical, legal and societal debate, but have already been addressed elsewhere [8,9]. Test characteristics and disease characteristics, however, are not entirely separable and may interact with one another as well as among themselves, as will be pointed out in the discussion.

The ethical debate may benefit from a better understanding of morally relevant differences between diseases. For national health care systems, policy-makers, healthcare professionals, physicians, companies or other stakeholders who are considering the offering or the regulation of susceptibility testing, it is important to be aware of differences between diseases or types of diseases, because susceptibility tests for different diseases may be connected with different ethical issues and may warrant different ethical evaluations. This poses challenges to an industry that is increasingly offering personal genome tests that consist of more and more susceptibility tests for more and more diseases rather than single susceptibility tests for single diseases. The insights offered in this paper are also anticipatory of future developments, and apply equally to susceptibility testing based on array technologies and to susceptibility testing based on exome or whole-genome sequencing technologies.

First, we will present and discuss a list of key disease characteristics that are crucial to the ethical appraisal of genetic susceptibility testing for multifactorial diseases. Then we will discuss susceptibility testing for three diseases, type 2 diabetes mellitus, age-related macular degeneration and clinical depression, and ethical issues that must be taken into account in the evaluation of such tests.

## Discussion

### 1. Disease characteristics and their associated ethical issues

We have conducted a review of the biomedical and bioethical literature and identified three existing normative frameworks, one for clinical genetics [10] and two for population genetic screening programmes, namely the Wilson and Jungner criteria [11] and the ACCE model [12], which take into account differences in disease characteristics and their impact on the ethical evaluation of testing. Not all disease characteristics or consequences of testing that are mentioned in the three frameworks, however, are applicable to genetic susceptibility testing for multifactorial diseases. For example, while characteristics such as the prevalence of the disease [13] or the population health impact [11] are relevant from a public health perspective, they are arguably less relevant for the ethical evaluation of susceptibility testing in individuals. Further, implications for relatives or availability of a follow-up plan [10] have been formulated in the context of clinical genetic testing for monogenic diseases and do not readily apply to testing for multifactorial diseases, where test results so far have been - and are likely to continue to be - of limited clinical validity and utility.

On the basis of an ethical analysis, we have identified four disease characteristics that are specifically relevant to the ethical evaluation of susceptibility testing: severity, actionability, age of onset, and the distinction between psychiatric and somatic diseases. These characteristics are morally relevant because they are directly connected with three of the four major bioethical principles: beneficence, non-maleficence and respect for autonomy [14]. Beneficence is the medical professional's duty to care, to do well, and to act in patients' interests. The principle of non-maleficence specifies that a medical professional should refrain from doing needless harm. Respect for a person's autonomy implies roughly that patients should be allowed to make their own voluntary, informed choices with regard to their health management. For the purposes of this paper, we leave aside the fourth principle, that of justice, because we focus on the justifiability of the provision of susceptibility testing *to an individual *rather than on societal, distributive issues that surround healthcare on a collective level. Below, we will discuss the four characteristics and describe how they give rise to ethical issues.

It is important to note at the outset of our discussion that internationally recognised guidelines on the provision of genetic testing [15,16] demand that medical supervision and genetic counselling are made available in genetic testing. The guidelines apply to genetic testing that is 'offered in a clinical context' [15] and genetic testing 'for health purposes' [16], respectively. It is therefore unclear whether these guidelines apply also to direct-to-consumer genetic testing or to genetic testing for purposes other than health. Both guidelines do specify that the form and the extent of genetic counselling "shall be defined according to the implications of the results of the test" [16] and "should be proportionate and appropriate to the characteristics of the test, the test limitations, the potential for harm, and the relevance of test results to individuals and their relatives" [15]. In our discussion, we will follow the idea that counselling requirments should be proportional and show how disease characteristics play a role in determining the appropriate level of counselling and medical care.

#### 1.1 Severity

Severity of a disease refers to the morbidity and mortality brought about by the disease. In clinical genetics, the severity of the disease affects the emotional, psychological and social impact of genetic test results. Disease severity therefore has consequences for the ethical requirement to offer good care (beneficence) [14] in the form of genetic counselling and psychological support. It is recommended that extensive pre- and post-test genetic counselling is mandated in all cases of severe hereditary disease [10]. One could argue that for severe diseases, where the anticipated psychological impact and the potential for harm are greater than for less severe diseases, as a general rule, more information, guidance and care are morally required.

The severity of a disease, however, is not always easy to determine: diseases tend to express differently from one individual to the next. High levels of phenotypic variability may cause genetic susceptibility test results to leave patients or consumers with substantial uncertainty with regard to the severity as well as the onset of the disease. These uncertainties may pose challenges for the provision of pre- and post-test information on personal genome testing and for its informed consent processes. Thus, disease severity affects the ethical issues of information, informed consent, genetic counselling, guidance and care.

#### 1.2 Actionability

There are potential harms involved in genetic testing, such as health risks (e.g. unnecessary follow-up), psychological risk (e.g. anxiety) and social risks (e.g. financial costs, discrimination). Therefore, in the ethical evaluation of genetic testing and screening services, a favorable balance between risks and benefits, i.e. between the principles of beneficence and non-maleficence, has traditionally been a central criterion [11]. The availability of therapeutic interventions constitutes a major benefit and a rationale [9,11] for genetic testing or screening. With part of medicine's focus shifting from cure to prevention over the last few decades [17], however, the notion of treatment has come to include other 'meaningful actionable options' [18], which were added primarily to include reproductive decision-making. The more contemporary ACCE framework introduces preventive options and actions as possible benefits of screening: it requires that there be "an effective remedy, acceptable action, or other measurable benefit" [12]. In the context of personal genome testing, with its wide variety of diseases tested for, actionability has become a more appropriate notion than treatability.

For susceptibility testing, *prevention *is argued to be one of the main purposes and potential benefit [2][[3,17]. Prevention may indeed constitute a benefit to both the individual and society and an argument for the moral acceptability of susceptibility testing. It should be noted that in order for preventive measures to be effective, compliance is essential. Compliance is generally promoted when preventive options are simple and morally or psychologically acceptable [19]. The daily taking of supplementary vitamins, for example, is likely to be accepted and carried out much more easily than more intrusive measures, such as medication with side-effects or a radical change of diet.

However, it is not at all clear that susceptibility testing contributes or will contribute to the prevention of multifactorial diseases [20]. If it does not or will not, its major rationale will lack ground, and therewith, it may not be able to reach the morally required balance of benefits and risks. An alternative rationale for susceptibility testing and potential benefit could be the personal utility [19] that consumers find in knowing their genetic information. It has been found that disease risk information obtained from a screening programme may serve psychological or personal purposes, such as relief from uncertainty [21], solace, the value of knowing. Whether personal utility on its own could provide a sufficient argument for the ethical acceptability of genetic susceptibility testing or personal genome testing, is still a topic for discussion [22].

#### 1.3 Age of onset

Through predictive genetic testing, it has become possible for individuals to know their genetic risk *before *the onset of symptoms of disease. Knowledge of genetic risk for late-onset disease may cause anxiety and distress. Not everyone wishes to obtain such knowledge [23].

One of the classic ethical principles within clinical genetics is therefore the right not to know, which is derived from respect for autonomy [24]. Especially in children the right not to know is a recurrent issue, because adverse psychological and social effects of predictive genetic testing for late-onset diseases may be severe in children [25], and also because children are considered incapable or less capable of making autonomous choices. It is generally agreed upon that in the absence of medical necessity, predictive genetic testing for late-onset diseases must be postponed until adulthood, when young adults have attained the ability to make autonomous decisions [25]. Exceptions are sometimes made when the clinical geneticist finds that the child or adolescent has sufficient cognitive ability to participate in decision-making and when the child's medical or other interests may weigh up against potential harms [21]. In clinical genetic practice, however, children and minors are more often refused than allowed predictive genetic testing for late-onset diseases [26]. These ethical principles may be equally applicable to genetic susceptibility testing for late-onset multifactorial diseases in children or minors.

The distinction between early-onset and late-onset of disease, however, cannot be so readily made in biological reality. In the pathogenesis of most multifactorial diseases, genetic and non-genetic risk factors act and interact over time to cause evolving stages of disease. In such a 'cascade model' of disease [27], the age of onset can not always be pinpointed straight-forwardly. Whereas the disease itself (e.g. symptoms of type 2 diabetes) may not become manifest before adulthood or even old age, certain risk factors (e.g. overweight) or early stages of disease (e.g. pre-diabetes) may be present already in youth. Naturally, genetic risk factors are present at conception. Since the first steps towards disease may already be made before birth or in early childhood, the notion of *onset *of disease could become less clear. In children or minors who already demonstrate certain risk factors or early stages of disease, genetic susceptibility testing for these diseases, even though they have traditionally been considered late-onset, could arguably be morally acceptable.

#### 1.4 Psychiatric/somatic distinction

There are at least three morally relevant differences between somatic and psychiatric diseases that may lead to ethical issues. Firstly, knowledge of genetic risks for psychiatric diseases could "undermine or weaken a person's sense of integrity and well-being" even before the appearance of symptoms [28]. Psychiatric diseases can change patients' perceptions of the world, their behaviors, desires, opinions and goals, their relationships, and who they are. The potentially greater psychological impact of genetic testing for psychiatric diseases may require a higher potential for medical benefit and higher standards for genetic counselling than those used for somatic diseases [28].

Secondly, psychiatric diseases are associated with stigma to a greater extent than somatic diseases [29,30]. Stigma may lead to social and societal problems, such as disturbed family or personal relations, or discrimination at the workplace or in the context of (health) insurance. The level of stigmatisation varies with the disease and partly determines the level of psychological and social risk involved [31]. Genetic susceptibility testing is thought capable of increasing rather than decreasing stigma associated with psychiatric diseases [32]. A high risk of stigma and subsequent psychological harm will increase the required potential for (medical) benefit as well as the need for good care and counselling.

Thirdly, it has been suggested that genetic testing itself or a positive test result may become a self-fulfilling prophecy, a 'trigger event' for the psychiatric disease tested for [33]. Moreover, the manner in which the disease is understood by the patient (e.g. "it is in my genes, therefore it is an inevitable aspect of myself") is likely to reflect on and modulate the development of the disease itself [34]. Thus, genetic testing could have unforeseen negative effects on the disease itself.

It is important to note here that the psychiatric/somatic distinction has a somewhat different status than the other disease characteristics. Modulated by actionability and severity, genetic testing for psychiatric diseases may be accompanied by a varying likelihood of adverse psychological consequences and thus bring the principle of non-maleficence into play to a varying extent. This way, disease characteristics may sometimes combine to lead to ethical issues. They should not be considered in isolation, but rather with an eye for their dynamic relations.

In conclusion, there is not yet much experience with (clinical) genetic testing for psychiatric diseases. Through personal genome testing, the practice of risk prediction for psychiatric diseases is currently taking shape, but the field and its ethical issues are largely unexplored. Due to the complexities in etiology, and the societal and psychological sensitivities that surround psychiatric diseases, genetic testing for such diseases requires a careful approach, with special attention to ethical issues.

### 2. Three diseases: how disease characteristics affect the ethical debate

We have identified four key disease characteristics that are relevant to the ethical evaluation of susceptibility testing: severity, actionability, age of onset, and the psychiatric/somatic distinction. In this section, we analyse how these disease characteristics influence the ethical issues surrounding susceptibility testing for three multifactorial diseases, namely type 2 diabetes, age-related macular degeneration (AMD) and clinical depression. These diseases are illustrative of both the disease characteristics and the main ethical issues surrounding susceptibility testing, whether it be offered in a clinical setting or directly-to-consumer. Our main question is whether and on what conditions it would be morally justifiable to offer individual susceptibility testing to children and adults. Key elements of the discussion are summarised in Table 1.

**Table 1:** Disease characteristics and their relation to ethical principles: Ethical issues to consider

Disease characteristic	Ethical principles	Relation characteristic/principle: Ethical issues to consider
Severity	Beneficence Non-maleficence	High severity → high potential for medical benefit, high risk of psychological harm

Actionability	Beneficence Non-maleficence	High actionability → high potential for (medical) benefit Moderate actionability → risk of psychological harm (e.g. victim-blaming or feelings of guilt) Low actionability → risk of psychological harm (emotional impact of test result)

Age of onset	Respect for autonomy	Late age of onset → right not to know in adults and children

Psychiatric/somatic	Non-maleficence	Psychiatric diseases: more risk of harm because of stigma; more risk of harm because of psychological impact

#### 2.1 Type 2 diabetes: variable severity and possibilities for early preventive intervention

Genetic susceptibility testing services for type 2 diabetes have already been made available directly-to-consumer both as targeted tests and as part of non-targeted personal genome tests [2,35]. While both physicians and consumers have been found to respond favorably to the idea of direct-to-consumer genetic testing for type 2 diabetes susceptibility [36], experts are not convinced of its current clinical validity and utility [37,38].

Type 2 diabetes is a somatic disease of varying severity. In its initial stages, it generally causes relatively mild symptoms, but on the long term, diabetes may cause kidney failure, blindness and neuropathy of the extremities, and it may cause premature death. As known non-genetic risk factors, such as overweight and pre-diabetes, are increasingly affecting the young [39], type 2 diabetes can no longer simply be considered a late-onset disease. There are both therapeutic options and well-established preventive strategies available for type 2 diabetes, for children as well as for adults, at the level of lifestyle improvements.

The variability of the severity of type 2 diabetes poses difficulties for the ethical evaluation of susceptibility testing for the disease. From a precautionary perspective, it could be argued that type 2 diabetes should be viewed as a severe disease and require high levels of genetic counselling and psychological support. However, empirical research has shown that on the short term genetic susceptibility testing for type 2 diabetes hardly causes any psychological harm or emotional impact at all [40,41], thus suggesting that consumers may not experience increased personal risk for type 2 diabetes as severe, and that requirements for counselling in order to prevent psychological harm may be less stringent. There may be discrepancies between the severity of a disease as perceived by medical professionals and the severity of the same disease as perceived by other publics. Questions regarding standards of severity may be interesting topics for further (ethical) research.

Further, the existence of preventive options for type 2 diabetes implies a potential for medical benefits to be obtained from susceptibility testing. Preventive options consist in general health recommendations, such as a healthy diet, regular physical exercise, weight loss, and smoking cessation. As a consequence, if false reassurance occurs, it may lead to harm. Individuals who are found to be at decreased risk may wrongly feel assured that they will remain free from disease, regardless of their lifestyles [42]. They may fail to understand that general health recommendations are relevant to the whole of the population, including low-risk subgroups. Low-risk individuals may ignore these recommendations and consequently put their health conditions at risk. The risk of false reassurance should be taken into account in the consideration of an offer of susceptibility testing for type 2 diabetes to adults or children.

The actionability of a test is not only a characteristic of the disease tested for, but is influenced by other factors, as well, notably the clinical validity of the test. As preventive measures in type 2 diabetes are identical to general health recommendations that apply to the entire population, it follows that it is unclear whether there is any use for susceptibility testing [37]. The actionability of susceptibility testing may nonetheless be twofold: it may motivate high-risk individuals to adhere to general health recommendations [42] and it may be used to target preventive strategies, guidance, care and monitoring by healthcare professionals, at those who are likely to be most in need. Any clinical utility - any potential for medical benefit - will only be realised on the condition of established clinical validity, which today has not been fulfilled.

Moreover, in the context of a limited or moderate clinical validity, preventive measures ought not to be too burdensome or too strongly associated with health risks, psychological or societal risks. When the benefits of preventive action are uncertain, the risks should be minimal in order to assure proportionality. In the case of type 2 diabetes, preventive measures are likely to be acceptable to most consumers: they may not always be easy to adopt, but they do not entail health risks or side effects. Adequate post-test counselling could help to improve the implementation of behavior change and thus actionability.

At the same time, the window of opportunity for preventive action is expanding at present. Given that childhood obesity may not only cause severe morbidity in the young [43], but is also an independent risk factor for adult obesity [44] and subsequent diseases (e.g. type 2 diabetes), it may be sensible to start the prevention of adult obesity or type 2 diabetes already in childhood. It is still unclear whether and what childhood interventions will prove effective [43,45], and whether genetic susceptibility testing for type 2 diabetes will gain clinical validity and will prove to be of added value over and above clinical risk factors for the identification of at-risk individuals [38]. If it can be established that early interventions yield levels of medical benefit that could not be otherwise attained, and that genetic testing can be useful for the targeting of interventions at subgroups of high-risk children, a favourable risk-benefit ration for testing in childhood might ensue. In that case it could become rational and morally justifiable to test children for their genetic susceptibility to type 2 diabetes. This will also depend on other ethical issues, such as psychological harms: at-risk children who do not adhere to lifestyle recommendations and develop the disease later in life may blame themselves or be blamed by others. Such 'victim-blaming' or feelings of guilt will not always be justified in the context of a multifactorial disease for which susceptibility testing is of moderate predictive ability: some at-risk individuals may develop the disease even if they take appropriate measures, whereas other at-risk individuals may not fall ill despite their failing to take preventive action. These risks must be weighed against the potential medical benefits.

In conclusion, while the age of onset may be difficult to determine for type 2 diabetes and the disease is rather severe, it is actionable. There is currently insufficient evidence to support an offer of genetic susceptibility testing for type 2 diabetes to children or minors. Susceptibility tests are presently of insufficient clinical validity and utility [38] in order to justify such an offer. For adults, however, it could be argued that in the absence of clear harms, the potential for benefit need not be great in order to justify genetic testing, on the condition, naturally, of adequate ethical safeguards such as adequate information provision, informed consent, quality assurance and privacy protection, and provided the test has a reasonable predictive ability. Stringent requirements for counselling may not be necessary to protect against psychological harm, but post-test counselling may be helpful to improve actionability and to protect against the risk of false reassurance.

#### 2.2 Age-related macular degeneration: very late onset

There have been optimistic reports on the feasibility of testing for genetic susceptibility to age-related macular degeneration (AMD) [46,47], and several personal genome testing services including AMD have already been put onto the direct-to-consumer market [2,48,49]. Genetic susceptibility tests for AMD are considered to be of substantial clinical validity, and are expected to eventually outperform and improve or replace existing prediction models [50].

AMD is a leading cause of vision loss worldwide [51]. It is a somatic disease of (very) late onset that generally progresses slowly. Although end-stage AMD may entail serious vision loss and brings along a fair amount of morbidity, the disease is not life-threatening. Furthermore, there are treatment options (e.g. laser therapy) and preventive options (specific vitamin supplements, smoking cessation, the wearing of sunglasses) available for AMD [52]. Although the preventive measures are safe, they do not seem to be very effective [53].

Given the late age of onset and the absence of opportunities for primary preventive action in childhood or adolescence, there are no medical reasons for childhood genetic testing. Therefore, it is preferable to postpone genetic testing for AMD until maturity, when young adults can make a more informed and autonomous decision whether or not to undergo testing and can provide informed consent. Thus, children's and minors' rights not to know their genetic risk can be protected.

As AMD tends not to strike until old age, psychological and social risks of genetic testing are likely to remain limited. Although there are no effective preventive options, given the availability of treatment options, the moderate severity and the (very) late onset of the disease, susceptibility testing for AMD will not give rise to very many ethical issues in consenting adults. Testing for AMD may become a morally acceptable practice in the clinic or on the direct-to-consumer personal genome testing market, depending largely on the ways in which further ethical issues are dealt with, such as quality assurance, information provision and informed consent, which are beyond the scope of this paper.

#### 2.3 Clinical depression: psychiatric disease, stigma, and psychological risk

Potential consumers have expressed high interest in genetic susceptibility testing for clinical depression [54]. They have also indicated to be interested in such testing for their children [55]. Several companies are offering susceptibility testing for clinical depression directly-to-consumer [56,57]. The clinical validity of susceptibility testing for clinical depression has not been established [58].

Clinical depression can present itself at any age [52]. While non-genetic risk factors for depression, such as emotional neglect or negative emotional experiences in early youth, high stress levels, major life events, and drug abuse, have been identified, they are notoriously difficult to avoid. As of yet, no feasible, effective primary preventive strategies have been established for depression, neither in children nor in adults. The disease varies in severity, but is generally considered to be relatively severe [52]. For severe diseases, it could be argued, susceptibility tests are morally required to be of sufficient clinical validity, because low or moderately predictive testing for severe diseases would leave the tested individual with high degrees of uncertainty in the light of a fearful medical scenario. This could cause psychological harm to the patient and so violate the principle of non-maleficence. Since, as of yet, the level of clinical validity in susceptibility testing for depression has not proven sufficient, the ethical acceptability of a testing offer is not clear.

Companies are increasingly marketing pharmacogenomic testing for drug response directly to consumers, also in the context of psychiatric diseases [2,59]. One company is offering a combination of susceptibility testing and pharmacogenomics testing for anti-depressant response [56]. Such combinations of susceptibility testing and pharmacogenomic testing are a new development on the direct-to-consumer genetic testing market. Theoretically, they may increase the actionability of the test and decrease the perceived severity of the disease tested for, and thus bring more benefit and less harm to individuals than would single susceptibility tests. On the other hand, however, this development may not be without risk. In the absence of evidence of clinical validity - and there is no such evidence for pharmacogenomic testing for antidepressant response [60] - potential for medical benefit is not likely to materialise. Sensitivities and risks surrounding genetic testing for psychiatric diseases should be weighed carefully.

It has been hypothesised that susceptibility testing for psychiatric diseases be made available to children [33]. Children who are tested to be at increased risk could be provided with 'pre-symptomatic interventions' [33], it is claimed, consisting of advice about avoiding environmental stress, or prophylactic medication. However, in the absence of feasible primary preventive options, predictive testing is not likely to yield any medical benefit for children. Genetic information about psychiatric diseases may have an unknown effect on the child's "developing sense of self and future prospects" [31], and may even become a self-fulfilling prophecy [33]. As long as clinical validity and utility are lacking, and taking into account moral considerations such as non-maleficence and the right not to know, there are no convincing reasons to justify the offering of genetic testing for clinical depression risk to children or minors.

In conclusion, in addition to its variable age of onset, relatively high level of severity, and unclear preventive options, clinical depression is characterised by its psychiatric rather than somatic nature. The disease is endowed with stigma, and knowledge about one's susceptibility may cause psychological risks (see section 1,4). Given the lack of 'actionability', genetic testing for depression may bring more harm than benefit onto persons. As long as uncertainty prevails regarding the psychological implications of genetic susceptibility testing for psychiatric diseases, a cautious approach may be warranted, even in consenting adults. More research into the psychological and social consequences of personal genome testing for depression and other psychiatric diseases will be needed.

## Summary

We have identified four disease characteristics that are relevant to discussions on the ethical issues surrounding genetic susceptibility testing for multifactorial diseases: severity, age of onset, actionability and the somatic or psychiatric nature of the disease. These characteristics are linked to important ethical principles and work together to affect the ethical debate. For example, the potential for adverse psychological and social consequences of genetic testing for late-onset diseases (harm) is greater in the context of diseases that are both severe and have no actionable options. In such cases testing poses greater ethical challenges.

As a general ethical rule of thumb, the likelihood and seriousness of possible harms, including psychological and social harms, should weigh up against the likelihood and magnitude of the potential (medical) benefits of testing. Severity and actionability are therefore relevant disease characteristics. Moreover, severity, actionability and the somatic/psychiatric distinction affect the requirements for good pre- and post test counselling, such that, for example, genetic susceptibility testing for psychiatric diseases will require careful psychological counselling. In children, the right not to know must be protected, which means that late-onset disease should not be tested for, unless there is a clear advantage (a positive benefit-risk ratio) for the child.

We have discussed these disease characteristics and the resulting ethical issues for three exemplary diseases, type 2 diabetes, age-related macular degeneration (AMD) and clinical depression. First, a broader perspective may be appropriate on the age of onset in type 2 diabetes to encompass accumulating risk factors and preclinical stages of disease throughout life. Genetic susceptibility testing for type 2 diabetes may eventually become acceptable even in children and minors, depending foremost on the clinical validity of the test, but also on the actionability of the test result, and on the manner in which 'age of onset' is conceptualised. Potential for medical benefit must be weighed against psychological harms and moral wrongs, such as infringements upon the right not to know. For adults, genetic susceptibility testing for type 2 diabetes may be acceptable under certain conditions. Second, we have described AMD as a less severe somatic disease of very late onset. We have concluded that not many ethical issues are to be expected from susceptibility testing for AMD in consenting adults, whether within a clinical context or on the direct-to-consumer market. Third, clinical depression is understood to be a psychiatric disease with a variable age of onset, a relatively high level of severity and unclear actionability. Genetic information on psychiatric diseases is associated with specific ethical issues, such as stigma and possible adverse psychological consequences, that warrant a very careful consideration of genetic testing for psychiatric diseases.

As a general conclusion we contend that a critical attitude is needed towards personal genome testing services that offer 'packages' of risk estimates for a multitude of multifactorial diseases simultaneously, because, as we have argued, different ethical issues apply to different diseases, depending on their characteristics. As some personal genome testing companies are offering genetic test results for a multitude of diseases that differ from one another with regard to the disease characteristics that we have identified [2], consumers are confronted with test results that vary in emotional impact and thus pose different requirements for pre- and post-test information and counselling. The ethical evaluation of such broad testing is therefore highly complex. Susceptibility tests for some diseases, such as AMD, can justifiably be offered within directly-to-consumer personal genome testing. For other diseases, on the other hand, it may not even be morally acceptable to include a genetic susceptibility test at all, or only on the condition of professional counselling. Finally, many tests may not be morally justifiable in the case of children, because of a late onset of the disease and a lack of actionability. Further research will be needed in order to establish a sensible subdivision of those broad 'packages' into clusters of diseases with similar characteristics, so as to allow for parallel ethical evaluations of clusters of susceptibility tests within a single personal genome test. Such parallel ethical evaluations should point out what clusters of tests may or may not justifiably be offered, and on what conditions.

When whole-genome sequencing becomes widely accessible to patients and consumers, and yield disease risks not only for multifactorial diseases but also for monogenic diseases, these problems are likely to increase even further. It will not be easy to conduct an overall ethical evaluation of personal genome testing on the basis of whole-genome sequencing, or to determine the appropriate and morally required level of genetic counselling, care and psychosocial support.

Although other aspects of genetic susceptibility testing, such as the more technical properties of the test or specific aspects of the context in which testing is offered, may be equally important to its ethical evaluation, we think that an understanding of ethically relevant disease characteristics will prove helpful for further ethical discussions on genetic susceptibility testing and personal genome testing for multifactorial diseases.

## Competing interests

The authors declare that they have no competing interests.

## Authors' contributions

All authors have contributed substantially to the conception and design of the manuscript. EB has drafted the manuscript. Both MS and AJ have critically revised it. All authors have read and approved the final manuscript.

## Pre-publication history

The pre-publication history for this paper can be accessed here:

http://www.biomedcentral.com/1755-8794/5/4/prepub
